# Advances in etiology, pathophysiology, diagnosis, and management of lymphedema: a comprehensive review

**DOI:** 10.3389/fmed.2025.1666522

**Published:** 2025-11-06

**Authors:** Tianqi Wu, Jun Pu, Qi Yao, Siying Chen, Youheng Yao, Suyan Chang, Riyun Yang, Jianhong Shen

**Affiliations:** 1 Medical Morphology Laboratory, Medical School of Nantong University, Nantong, China; 2 Department of Neurosurgery, Affiliated Hospital of Nantong University, Nantong, China; 3 Department of Histology and Embryology, Medical School of Nantong University, Nantong, China

**Keywords:** lymphedema, primary lymphedema, breast cancer-related lymphedema, lymphoscintigraphy, indocyanine green lymphography, magnetic resonance lymphangiography, lymphaticovenous anastomosis, liposuction

## Abstract

Lymphedema is a chronic disorder of impaired lymphatic transport that leads to fluid accumulation, fibrosis, and adipose expansion. It presents as primary disease, caused by genetic defects in lymphatic development, or as secondary disease after surgery, radiotherapy, infection, trauma, or malignancy. Recent studies have broadened the genetic basis of primary forms and clarified host and treatment-related risks for secondary forms. Mechanistic insights show that lymphatic stasis drives inflammation, fibrosis, and hypoxia, which remodel the microenvironment and reinforce lymphatic failure. Advances in imaging, including lymphoscintigraphy, indocyanine green lymphography, and magnetic resonance lymphangiography, enable earlier and more accurate diagnosis. Conservative treatment with complete decongestive therapy remains standard, while microsurgical techniques such as lymphaticovenous anastomosis and vascularized lymph node transfer expand options. Emerging pharmacologic strategies that target immune and fibrotic pathways show promise. This review summarizes current progress and highlights opportunities for precision interventions to improve outcomes.

## Graphical Abstract

**Figure:**
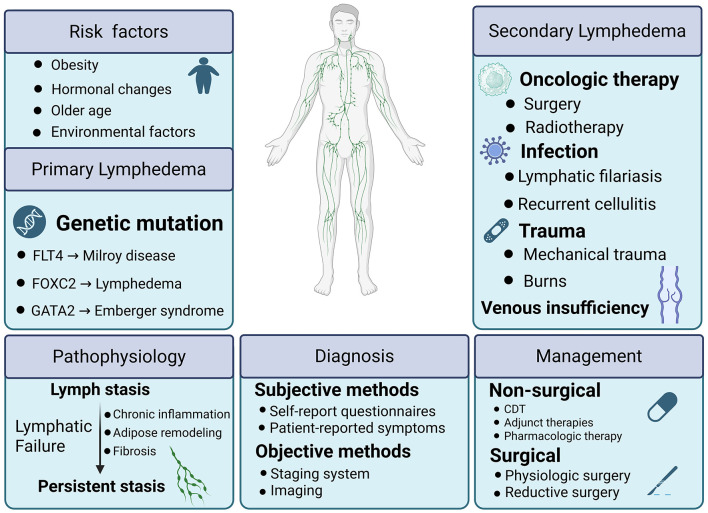

## 1 Introduction

The lymphatic system is unique to vertebrates and functions as a drainage of excess interstitial fluid, fat absorption and immune surveillance. It consists of lymphatic vessels, lymphatic tissues, and lymphatic organs. Unlike blood vessels, lymphatic vessels do not form a closed circulation but operate as a unidirectional system, with lymph flowing centripetally toward the heart. The system begins with initial lymphatic vessels, followed by precollectors, collectors, and trunks. These vessels are composed of a single layer of lymphatic endothelial cells (LECs) connected by anchoring filaments, which open lymphatic junctions when tissue pressure increases, facilitating the absorption of macromolecules, cells, and interstitial fluid. Lymph, absorbed from the interstitial spaces, contains proteins, water, fatty acids, salts, white blood cells, microorganisms, and cellular debris. It enters the lymphatic vessels, where it is transported through collectors and trunks before being filtered through lymph nodes. Ultimately, the lymph reenters the venous circulation. There are two distinct systems of lymphatic drainage: the superficial system, responsible for draining the skin and subcutaneous tissues, and the deep system, which drains the tissues located beneath the fascia. The interconnection between these two systems is facilitated by perforating vessels penetrating the fascia.

Lymphedema arises when there is an imbalance between the microvascular filtration rate of the capillaries and venules, and the capacity of the lymphatic drainage system to remove the excess interstitial fluid. This results in the accumulation of interstitial fluid in the tissue spaces, particularly in the subcutaneous tissue, leading to the manifestation of swelling. The primary symptom of lymphedema is swelling of the affected area, which can sometimes be severe and deforming, causing significant discomfort and impacting mobility. Depending on the cause, lymphedema can be divided into primary and secondary lymphedema.

Primary lymphedema results from an intrinsic fault in the lymphatic vessels such as Milroy's disease and Meige's disease, characterized by the underdevelopment or absence of the lymphatic system, resulting in a diminished ability to absorb interstitial fluid.

Secondary lymphedema, also known as acquired lymphedema, results from damage to the lymphatic vessels or nodes due to factors such as surgery, radiation, trauma, or infection caused by lymphatic filariasis. In addition to these causes, secondary lymphedema can also arise from chronic venous insufficiency, obesity, environmental factors (e.g., podoconiosis), and even self-harm. Chronic venous insufficiency leads to impaired venous return, which negatively affects lymphatic drainage. Obesity, through excess adipose tissue, can exert pressure on lymphatic vessels, hindering their function. Podoconiosis, caused by prolonged exposure to certain soils, can also disrupt the lymphatic system. Lymphatic gene dysfunction can also affect immune function leading to infection which can influence cancer development. Secondary lymphedema is a prevalent kind of lymphedema that impacts a substantial number of individuals globally. It is commonly associated with various tumors, especially in gynecologic oncologic surgery, where the incidence of lymphedema is as high as 20% (1). Given the profound impact on patients' quality of life and the complexity of its mechanisms, lymphedema has become an important focus of both basic and clinical research. This review aims to summarize recent advances in etiology, pathophysiology, diagnosis, and management, while highlighting translational opportunities for improved care.

## 2 Etiology of lymphedema

Lymphedema arises either from primary defects in lymphatic formation or from secondary injury to lymphatic vessels and nodes (2). The underlying etiology dictates the downstream pathophysiologic cascade. Persistent lymph stasis leads to chronic inflammation, progressive fibrosis, and adipose tissue remodeling (3). These processes provide the foundation for prevention, risk stratification, and management strategies. Recent advances have broadened the genetic spectrum of primary lymphedema and refined the quantification of both treatment-related and host-related risks for secondary disease, although a substantial proportion of primary cases remain genetically unexplained (4).

### 2.1 Primary lymphedema

Primary lymphedema results from congenital or hereditary defects of the lymphatic system. To date, more than 30 genes have been associated with abnormal lymphangiogenesis, defective lymphatic valve formation, or impaired lymphatic vessel contractility (5). The monogenic causes and phenotypes of lymphedema, including inheritance patterns and clinical features, are summarized in Table 1, whereas biomarkers and molecular pathways are presented in Table 2. These discoveries have been driven by next-generation sequencing, which has revealed both coding and regulatory variants.

**Table 1:** Monogenic causes and phenotypes of lymphedema.

**Genes**	**Function**
Fms related receptor tyrosine kinase 4 (FLT4/VEGFR3)	High-affinity receptor for VEGFC (157); essential for lymphatic endothelial cell survival, proliferation, and lymphangiogenesis (158).
Vascular endothelial growth factor C (VEGFC)	Primary lymphangiogenic growth factor (159); binds and activates VEGFR3 to stimulate lymphatic vessel sprouting and growth (160).
Forkhead box C2 (FOXC2)	Transcription factor regulating lymphatic valve formation, patterning, and inhibition of pericyte coverage on lymphatic vessels (161).
GATA binding protein 2 (GATA2)	Transcription factor necessary for lymphatic valve maintenance and the expression of cell junction molecules (162, 163).
Collagen and calcium binding EGF domains 1 (CCBE1)	Enhances the bioactivity and signaling of VEGFC (164); critical for lymphatic endothelial cell budding and migration (165, 166).
A disintegrin and metalloproteinase with thrombospondin motifs 3 (ADAMTS3)	Protease that processes and activates VEGFC (167); expressed in pro-lymphangiogenic stromal cells (168).
EPH receptor B4 (EPHB4)	Receptor involved in cell repulsion (169); interacts with EFNB2 to regulate lymphatic valve formation by spatially inhibiting Erk signaling (170).
Piezo type mechanosensitive ion channel component 1 (PIEZO1)	Mechanosensitive ion channel (171); dysfunction can lead to lymphatic vascular defects and lymphedema (172, 173).
Protein tyrosine phosphatase non-receptor type 14 (PTPN14)	Involved in cell growth and differentiation (174); associated with lymphatic development and lymphedema (175).
Gap junction protein gamma 2 (GJC2)	Genetic variations associated with secondary lymphedema risk (176).
FAT atypical cadherin 4 (FAT4)	Regulates planar cell polarity and collective cell migration during lymphatic vessel development (177).
Kinesin family member 11 (KIF11)	Motor protein; mutations cause syndromes featuring microcephaly and lymphedema (178–180); role in lymphatic function is indirect (178).
Gap junction protein alpha 1 (GJA1)	Forms connexin 43 gap junctions; implicated in cell communication within the lymphatic vasculature (research ongoing).
SRY-box transcription factor 18 (SOX18)	Master transcription factor initiating lymphatic endothelial cell differentiation and development (181, 182).
Protein tyrosine phosphatase non-receptor type 14 (PTPN14)	Regulates cell adhesion and growth (175).
Dachsous cadherin-related 1 (DCHS1)	Interacts with FAT4; involved in planar cell polarity signaling for lymphatic valve morphogenesis (183).
Vascular endothelial growth factor C (VEGFC)	Primary ligand for VEGFR3; master stimulator of lymphatic vessel growth (lymphangiogenesis) (184).
Forkhead box C2 (FOXC2)	Transcription factor critical for lymphatic valve formation and vessel maturation (161).
Gap junction protein gamma 2 (GJC2)	Forms connexin 47 gap junctions; mutations linked to hereditary lymphedema (185, 186).

**Table 2:** Biomarkers or pathways of lymphedema.

**Biomarkers**	**Function summary**
Hepatocyte growth factor (HGF)/c-MET pathway	Promotes lymphatic endothelial cell growth, migration, and protects against radiation-induced lymphatic injury (187, 188).
Angiopoietin 2 (ANG2)/TIE2 signaling	Regulates lymphatic vessel maturation, stability, and permeability. Imbalance leads to vascular leakage (189, 190).
Transforming growth factor beta 1 (TGF-β1)	Key driver of tissue fibrosis and chronic inflammation in secondary lymphedema (26).
Interleukin 6 (IL-6)	Pro-inflammatory cytokine; elevated in lymphedema tissue, contributing to chronic inflammation and fibrosis (30).
C-C motif chemokine ligand 2 (CCL2)	Recruits monocytes/macrophages to the lymphedematous limb, promoting inflammation and tissue changes (191).
Lymphatic vessel endothelial hyaluronan receptor 1 (LYVE1)	Receptor for hyaluronic acid (192); a specific marker for lymphatic endothelial cells (192).
Podoplanin (PDPN)	Transmembrane glycoprotein critical for lymphatic development, separation from blood vasculature, and function (193, 194).
Proximal homeobox protein 1 (PROX1)	Master control transcription factor for lymphatic endothelial cell fate and maintenance (195).

Canonical clinical entities include Milroy disease, caused by mutations in FLT4/VEGFR3, and lymphedema–distichiasis syndrome, associated with FOXC2 mutations. Emberger syndrome, caused by GATA2 haploinsufficiency, links primary lymphedema with hematological malignancies (6, 7). More recently, novel variants in PROX1 and HGF have been reported, expanding the genetic landscape (8, 9).

Despite progress, most patients with primary lymphedema remain variant-negative with current testing, suggesting further undiscovered genes or complex regulatory mechanisms (10). Phenotypic variability is also striking: onset may occur at birth, adolescence, or adulthood, and severity is strongly modified by age, hormonal status (puberty, pregnancy), and body mass index (BMI) (11). High BMI in particular has emerged as a consistent risk factor for earlier onset and worse outcomes, highlighting the interplay between genetic predisposition and lifestyle and environmental modifiers (12, 13).

### 2.2 Secondary lymphedema

Secondary lymphedema is an acquired condition, most commonly occurring after oncologic therapies. Infections such as lymphatic filariasis remain a leading cause worldwide (14). In cancer survivors, both surgery and radiotherapy constitute the principal risk factors (15). The extent of lymph node dissection, particularly in the axillary or pelvic basins, is directly associated with lymphedema risk, with higher nodal counts predicting increased incidence and severity (16, 17). Radiotherapy contributes by inducing fibrosis and obliteration of lymphatic pathways; when combined with surgery, risk is synergistically elevated (18).

Host factors further modify risk. Obesity independently increases both incidence and severity, while older age and recurrent cellulitis are additional contributors. Recognizing these modifiers, validated risk prediction models have emerged: for example, a five-factor model (age, BMI, breast density, nodal burden, and axillary dissection) accurately stratifies 2-year lymphedema-free survival in breast cancer cohorts (19). Prospective data in gynecologic oncology confirm similar long-term predictors (20).

Beyond oncology, lymphatic filariasis (LF) remains a significant global health problem. Recent estimates suggest that tens of millions of individuals suffer from lymphedema as a chronic sequela of filarial infection, with global figures ranging up to 40 million affected cases (21). This highlights the persistent burden of infection-related secondary lymphedema, particularly in endemic regions.

## 3 Pathophysiology of lymphedema

The pathogenesis of lymphedema is increasingly recognized as a self-perpetuating cascade. Lymphatic stasis triggers a CD4? T-cell–driven inflammatory response that promotes extracellular matrix fibrosis and progressive tissue stiffening. In parallel, adipose tissue undergoes metabolic reprogramming, marked by dysregulated lipid metabolism and altered immune signaling. These events unfold within a hostile microenvironment characterized by hypoxia, pro-inflammatory cytokines, and tissue injury secondary to surgery or radiotherapy. The interplay of these factors drives lymphatic pump failure and the progressive rarefaction of lymphatic vessels (2, 22, 23). Foundational human and murine studies have shown that CD4? T cells become activated in regional lymph nodes and subsequently home to the skin after lymphatic injury. Depletion or functional blockade of these cells prevents lymphedema and improves lymphatic transport, establishing adaptive immunity as a causal driver rather than a bystander. Mechanistically, Th2 polarization with interleukin-4 and interleukin-13 suppresses lymphangiogenesis and promotes extracellular matrix deposition (24). Translationally, topical calcineurin inhibition with tacrolimus attenuates pathogenic T-cell signaling, reduces edema and fibrosis, and promotes collateral lymphangiogenesis in preclinical models, supporting immune modulation as a disease-modifying rather than purely symptomatic approach (25). Converging evidence identifies transforming growth factor-β1 (TGF-β1) as a central profibrotic hub that links inflammation to myofibroblast activation, collagen and elastin deposition, and extracellular matrix cross-linking. Pharmacologic or genetic inhibition of TGF-β1 signaling attenuates fibrosis and lymphatic dysfunction in experimental lymphedema (26). Hypoxia pathways intersect this axis: in both human and murine tissues, lymphatic HIF-2α expression is reduced while HIF-1α is elevated. Restoration of HIF-2α/TIE2 activity stabilizes lymphatic endothelium and mitigates pathological remodeling, implicating oxygen sensing as an upstream regulator of matrix–vessel crosstalk (22, 27). A defining late feature of lymphedema is adipose expansion, termed lymphedema-associated adipose tissue, characterized by up-regulation of inflammatory cytokines and lipid-handling genes such as PPARγ and C/EBPα. Multi-omic and clinical studies indicate that inflammation precedes and drives fat deposition, with interleukin-6 emerging as a key immunometabolic node that modulates adipose homeostasis in lymphedematous limbs (28–30). These biological processes are determined by the local tissue microenvironment. Surgical or radiotherapy-induced injury, together with persistent cytokine signaling, establishes an adverse niche characterized by fibrosis, pathological adipose accumulation, hypoxia, and increased extracellular matrix stiffness. This remodeled microenvironment reduces lymphatic vessel compliance and intrinsic contractile function, impairs lymphangiogenesis, and perpetuates lymph stasis. The resulting feed-forward interaction between stasis, chronic inflammation, and fibroadipose remodeling closes a pathogenic loop that helps explain the clinical refractoriness observed once fibroadipose changes predominate (31–33). Contemporary expert reviews have synthesized these mechanistic insights into an integrated framework that supports multi-pronged therapeutic strategies. Such approaches include modulation of immune and cytokine pathways, targeting of fibrotic remodeling and extracellular matrix dynamics, and interventions aimed at the microenvironment and oxygen-sensing mechanisms. When combined with early detection, these strategies hold the potential to disrupt the pathogenic cycle before irreversible fibroadipose remodeling becomes established (31, 34).

## 4 Diagnosis: objective methods and subjective methods

The risk of lymphedema peaked at 12–30 months after surgery (35). The lymphatic function is regulated by a variety of factors, including chronic inflammation, tumors, external stimuli (eg., radiation), age, obesity, and metabolic dysfunction. These factors will affect the occurrence and development of lymphedema. The primary presentation of lymphedema involves the buildup of interstitial fluid rich in proteins within the subcutaneous and subfascial tissues which triggers the initiation of an inflammatory response. The retended fluid exacerbates tissue fibrosis, deposition of fat, and formation of scars (36). This fibrotic development is mediated by the synthesis of pro-fibrotic cytokines by Th2 cells (such as IL-4, IL-13, and TGF-β1). These cytokines can affect the survival, proliferation, and migration of lymphatic endothelial cells (37). Lymphedema is known to elicit systemic alterations beyond the confines of the affected limb. Research has shown that collagen accumulation and the presence of CD4+ cells can be observed in tissues unaffected in the patients of lymphedema (38). Patients with the acute phase of lymphedema are susceptible to the development of cellulitis (erysipelas) and there may be a further decline in physical function. Psychological effects may also occur, leading to a lower quality of life (QOL) (39). One study analyzed the association between breast cancer-related lymphedema (BCRL) and cellulitis incidence and mortality in the National Health Insurance database in Taiwan. The results showed that the incidence and mortality rates of cellulitis were significantly higher in patients with BCRL than in patients without BCRL, and there was a significant correlation between the three (40). Therefore, early diagnosis and treatment play an important role in delaying disease progression and improving prognosis.

The diagnosis of lymphedema is based on the patient's pathography, physical examination, and ancillary tests such as lymphography and tissue biopsy (41). Associated risk factors such as previous surgery with nodal dissection, radiation therapy, infection, malignancy, family history of congenital lymphedema, and trauma should be taken into consideration during the diagnostic process (42). Depending on the etiology, further tests may be carried out to assess the severity and cause of the lymphedema. Imaging techniques for visualization of the lymphatic system are less well developed than those for imaging of blood vessels for the reason that the lymphatic system is more difficult to be visible (43). Technological advances in biomedical imaging have opened new possibilities in the diagnosis of lymphedema (44). Effective diagnostic measures play a crucial role in the identification and prevention of this disease, as well as aiding in the process of treatment and rehabilitation (45). The precise identification of a patient's health status together with the prompt detection and intervention at an early stage are essential ways of rehabilitation and preventing potential complications and optimization of therapy intervention can be achieved through the diagnosis of early-onset lymphedema. Multiple methods are available for the quantification of physiological alterations that manifest in lymphedema (46). Different diagnostic procedures include inherent limitations which often entail the use of many approaches in combination. A reasonable staging system plays an important role in the diagnostic process of lymphedema which promotes consistency and comparability in the diagnosis. Though imaging techniques for visualization of the lymphatic system are objective and standardized, subjective methods are implemented at the same time due to their convenience, low cost, and supplementary function. Subjective ways like self-report questionnaires can promote rapid diagnosis and facilitate the evaluation of treatment outcomes. Patients often report symptoms such as pain, heaviness, and discomfort when having or in the developing process of lymphedema. Combining subjective symptomatic and quality-of-life self-reports with objective measures is of great help in defining the level of lymphedema (47).

### 4.1 Objective methods for lymphedema

#### 4.1.1 Staging systems of lymphedema contribute to the uniform diagnosis

The staging system is a crucial component in the management of lymphedema and assists with the standardization of diagnostic procedures and treatment protocols. The implementation of standardized staging criteria for lymphedema in clinical practice serves several important purposes. A universal staging system promotes consistency and comparability in the diagnosis process across different medical professionals and institutions, minimizing regional variations, and thereby ensuring uniformity and reliability in the obtained results. The utilization of a uniform staging system helps to mitigate diagnostic discrepancies that may arise due to subjective factors, enhancing the accuracy and objectivity of the diagnostic process. Additionally, the adoption of standardized criteria facilitates physicians' comprehension of the condition, enabling effective communication with patients, formulation of appropriate treatment plans, and ultimately enhancing the credibility and reliability of medical outcomes (45, 48–50).

An ideal staging system should be comprehensive, reproducible, and correlate with imaging and clinical manifestations. While there exist many staging methods in use, the most often utilized one is recommended by the International Society of Lymphedema (ISL), which classifies lymphedema into four levels of severity, namely stage 0 to stage III. The corresponding clinical manifestations and sorting criteria are shown in Table 3, which has been adapted from ISL (2023) (51).

**Table 3:** Clinical manifestations and sorting criteria.

**Stage**	**Clinical manifestation**
0	Latent or subclinical condition, subtle swelling, impaired lymph transport, having subjective symptoms
I	Protein-rich fluid accumulation; pitting edema may occur
II	Fibrotic changes; persistent swelling; poor response to limb elevation
III	Severe lymphedema with marked skin thickening, fat deposition, and fibrosis

While the staging approach proposed by ISL demonstrates reproducibility, it lacks adequate inclusion of the physiological characteristics associated with lymphedema (49). It solely refers to the physical state of the extremities and lacks the ability for spatial distribution. Thus, a more detailed and inclusive classification is needed when special circumstances arise (45).

When it comes to the description of trunk lymphedema linked with breast cancer, the Pittsburgh Trunk Lymphedema Staging System (PTLSS) is proved to be a validated staging system which is a reliable method for assessing the extent of lymphedema in the whole trunk (52). Other staging systems and their corresponding methods used to assess the existence and severity of lymphedema are listed in Table 4. Various staging methods own their own set of pros and limitations. The advent of novel technology necessitates and propels the development of fresh staging systems.

**Table 4:** Staging systems.

**Staging system**	**Key features**	**Advantages**	**Limitations**
ISL staging (51)	Four stages (0–III) based on clinical severity; Severity classification: In each period, according to the degree of volume exceeding the standard, it is divided into mild (>5%−20%), moderate (20%−40%) and severe (>40%)	Simple, widely used, reproducible	Relies mainly on physical signs; lacks physiological detail; Dysfunction, quality of life or lymphedema in the head, torso and other parts were not evaluated
Lymphoscintigraphy staging (196)	Classification based on lymphatic flow obstruction	High accuracy; validated	Radiation exposure; poor spatial/temporal resolution (197)
ICG lymphography (198, 199)	Visualizes superficial lymphatic flow patterns (200)	Non-ionizing, real-time, high temporal resolution (201)	limited tissue penetration (202), potential off-label status of intradermal injection depending on jurisdiction

#### 4.1.2 Lymphedema imaging: major technologies

Among various diagnostic technologies, lymphoscintigraphy is widely recognized as the benchmark imaging modality utilized in the diagnosis of lymphedema (53). Lymphoscintigraphy can accurately visualize and delineate the lymphatic system, as well as identify the precise location of sentinel nodes. This technology offers comprehensive and precise data about the diagnosis and classification of lymphedema affecting the extremities, as well as its corresponding severity assessment (54). During the process, lymphoscintigraphy entails the administration of a tracer dye into the distal extremity, where it is afterward absorbed by the lymphatic vasculature (55). It allows for the examination of lymph-node uptake in the groin and axillary regions, as well as the identification of potential dermal backflow, collateral pathways, and delayed nodal uptake, with visualization of the popliteal or epitrochlear lymph nodes (39). Lymphoscintigraphy serves as a valuable indicator for BCRL (56) and is an efficacious approach for diagnosing and confirming cases of primary lymphedema (39). The utilization of this diagnosis modality has proven to be beneficial in managing limb edema cases where etiology is not well understood or in patients who are suspected to have lymphedema (57). The procedure is characterized by low levels of radiation. However, it is contraindicated for individuals who are pregnant or breastfeeding. Indocyanine green (ICG) lymphography is a valuable lymphatic imaging technique that can visualize superficial lymph flow in real time without exposing the patient to radiation (58). It has established itself as a cornerstone technique in many clinical settings, with its principal applications encompassing pattern classification and surgical planning (59–62). The procedure is simple and convenient to execute, rendering it valuable for real-time comprehension of the lymphatic system's state (63). ICG dye was administered at three distinct sites in the distal arm or leg: two interdigital injections and one injection at the volar wrist or posterior to the medial malleolus. An immediate scan was conducted to assess lymphatic pump velocity and function. A follow-up scan, performed 6 h later, visualized the dermal backflow (DB) pattern, which is diagnostic for lymphedema and indicative of disease severity (64). It shows the DB pattern on both the thigh and lower leg regions demonstrating a high level of sensitivity and specificity in the identification of aberrant lymph circulation. According to the visibility of lymphatics and DB extension, the ICG lymphography pattern was categorized as linear, low enhancement (LE), distal DB, or extended DB in bilateral lymphedema. Each has different patient characteristics (65). ICG is conventionally injected in the distal leg or arm. In cases when lymphatic activity is insufficient in the distal limb, the precise location of lymphatic vessels cannot be ascertained. This is true even if lymphatic vessel function is satisfactory in the proximal limb. Consequently, injecting the dermal leg may prove to be futile (66). Except for the distal part of the limbs, the first web space of the foot, the lateral ankle, and the lateral thigh are chosen to be the injection site (67). The aforementioned study revealed that Multi-lymphosome ICG lymphography demonstrates superior performance in the given scenario. The use of this method facilitates enhanced identification of functioning lymphatic vessels during lymphatic venous anastomosis (LVA), thus leading to improved surgical results (66–68). However, ICG lymphography is limited by its shallow detection depth and low spatiotemporal resolution. In contrast, Protein@Cyanine-based NIR-II lymphography overcomes these limitations, enabling the highly sensitive visualization of lymphedema and tumor lymphatic metastasis, thereby presenting a promising strategy (69).

Magnetic resonance lymphangiography (MRL) has become a new non-invasive method capable of delivering high-resolution three-dimensional images of an entire limb, with enough detail to identify individual lymphatic channels and regions of dermal backflow (70). MRL is neither sensitive nor specific for lymphedema but it is capable of visualizing preclinical alterations in lymphatic flow thus contributing to the early diagnosis of lymphedema and can evaluate other causes of limb swelling (71–73). MRL has demonstrated satisfactory outcomes in differential diagnosis, quantitative classification of disease severity, and optimal treatment planning (74). Dynamic contrast-enhanced MR lymphangiography is increasingly used for 3D deep anatomy (75). Dynamic contrast-enhanced magnetic resonance imaging via intranodal, intrahepatic, and intramesenteric routes enables the direct visualization of the central lymphatic system from the inguinal region to the venous angle (76). Dynamic contrast-enhanced magnetic resonance lymphangiography serves as the reference standard for diagnosing a range of thoracic lymphatic diseases, such as traumatic chylothorax and plastic bronchitis (77).

CT is an objective method to assess patients undergoing lymphadenectomy (78). CT-based quantitative assessments can offer objective volume measurements and detailed information about the structural characteristics of subcutaneous tissue (79). During CT scanning, patients were instructed to keep an anatomically neutral position with both arms fully extended. The CT scanned both limbs simultaneously, from the point where the legs separate to 5 cm above the lateral malleolus, or from the distal ends of the clavicles to the wrist crease (80). The study found that assessment of subcutaneous fat thickness using CT lymphangiography is useful for screening lymphedema at an early stage (81) and is beneficial for planning microsurgical therapies (82).

Ultrasound is a readily accessible bedside technique for evaluating dermal thickness, echogenicity, and tissue stiffness in patients with lymphedema. Nevertheless, its diagnostic performance is generally inferior to that of lymphoscintigraphy, indocyanine green lymphography, or magnetic resonance imaging. Owing to nonspecific B-mode findings and operator dependence, ultrasound is best regarded as a complementary rather than a stand-alone modality for lymphedema diagnosis (44, 83). Besides conventional B-mode ultrasound, advanced techniques such as strain (compression) elastography have also been applied in recent years to provide additional contrast in tissue stiffness. For example, Demirci et al. (84) used strain elastography to demonstrate significant differences in strain parameters between affected and unaffected limbs. Also, Yang et al. (85) developed a 2D registration-based strain imaging method for arm lymphedema and showed higher strain values in affected arms vs. contralateral arms. Furthermore, in a recent integrative study from Jeon et al. (86) strain elastography combined with multi-frequency bioimpedance was used to classify tissue stiffness phenotypes in lymphedema limbs and correlated with clinical severity. In lymphedema, strain elastography may help detect early microstructural changes such as incipient fibrosis or stiffening and monitor response to therapy by quantifying subtle changes in tissue elasticity over time.

Different approaches possess unique qualities. ICG lymphography is limited to visualizing superficial lymphatic flows up to a depth of 1.5 cm. In contrast, lymphoscintigraphy and magnetic resonance lymphography are better suited for evaluating deep lymphatic flows (65). However, Lymphoscintigraphy exposes patients to ionizing radiation and has poor spatial and temporal resolution. Each examination has merits and demerits, and we should combine several examinations to evaluate the lymphatic conditions accurately (67, 87). For instance, MRL can serve as a complementary method to be used alongside ICG-L in preoperative evaluations for LVA. This is because it is less effective at identifying lymphatic vessels in the initial stages of lymphedema when lymph stasis or lymphangiectasia are not present (88). Ultrasonography provides a noninvasive and non-ionizing diagnostic technique for patients with lymphedema by assessing the shear wave speed of subcutaneous tissue and visible dermal structure (89, 90). Other objective evaluations of lower extremity lymphedema including dual-energy X-ray absorptiometry (DXA) (91), and imaging biomarkers (92), serve the diagnostic procedure of lymphedema in different ways. The advantages and limitations of some of the technologies are summarized in Table 5.

**Table 5:** Imaging technologies.

**Imaging technique**	**Advantages**	**Limitations**
X-ray lymphography	Provides deep tissue penetration and direct visualization of lymphatic channels	Invasive; rarely used in current practice
Lymphoscintigraphy	Considered the gold standard; high accuracy for detecting lymphatic obstruction; useful for staging	Involves ionizing radiation; poor spatial and temporal resolution
Ultrasonography	Non-invasive; no radiation; reliable for assessing soft tissue changes and dermal structure	Limited to superficial tissues; operator dependent
Magnetic resonance lymphangiography (MRL)	Non-invasive; high-resolution 3D imaging; allows visualization of lymphatic channels and dermal backflow	Expensive; variable sensitivity and specificity; limited availability
Computed tomography (CT)/CT lymphangiography	Provides detailed anatomical localization and quantification of subcutaneous tissue changes	Radiation exposure; limited temporal resolution
Near-infrared fluorescence lymphography (NIRF-LI/ICG lymphography)	Clinically approved (203, 204); Real-time (201), dynamic visualization (63); no radiation (205); high sensitivity for superficial lymph flow (200)	Limited penetration depth ( ≤ 1.5 cm); requires dye injection
Dual-energy X-ray absorptiometry (DXA)	Objective and quantitative assessment of limb volume and composition	Limited application; less specific for lymphatic dysfunction
Optical/molecular imaging and biomarkers	Provide functional and molecular-level insights into lymphatic health; potential for early detection	Mostly experimental; not yet standardized for routine clinical use

### 4.2 Subjective methods for lymphedema diagnosis

Compared with the objective methods, the subjective method bears its advantages. It is convenient and cost-effective to perform. Patients who need a long-term rehabilitation process, and higher frequency diagnosis require simpler diagnostic methods since the objective methods can be expensive and complicated if employed frequently. Some patients with limited time are in favor of the subjective assessments compared to the more time-consuming objective measures, enabling more time to be dedicated to treatment. Moreover, since lymphedema is complicated and differs among patients, objective methods alone may not be performed smoothly which requires subjective methods to play a crucial role in the diagnosis process.

Generally, patients suspected of lymphedema complain about subjective symptoms such as a feeling of heaviness, numbness, or tingling. To get insights into these symptoms, self-report tools such as lymphedema-specific questionnaires consistent with symptoms of lymphedema can promote rapid diagnosis and facilitate evaluation of treatment outcomes (93). Lymphedema Symptom Intensity and Distress Survey Arm (LSIDS-A) is a reliable and valid instrument to assess arm lymphedema and its multidimensional symptoms which provides valuable information that can be used to inform clinicians and enhance patient care (94). When involved lymphedema occurs in lower limbs, the Lymphedema Symptom Intensity and Distress Survey-Lower Limb (LSIDS-L) is a valid tool for detecting and quantifying symptoms of patients with lymphedema (95). A similar tool can be used to evaluate symptoms related to the head and neck (96). Subjective clinical measures are often based on medical history and physical examinations (85). During subjective measures, the unaffected side provided a baseline for comparison, comparing changes over time (97) and assessing patients' subjective symptoms (98).

**Figure 1:**
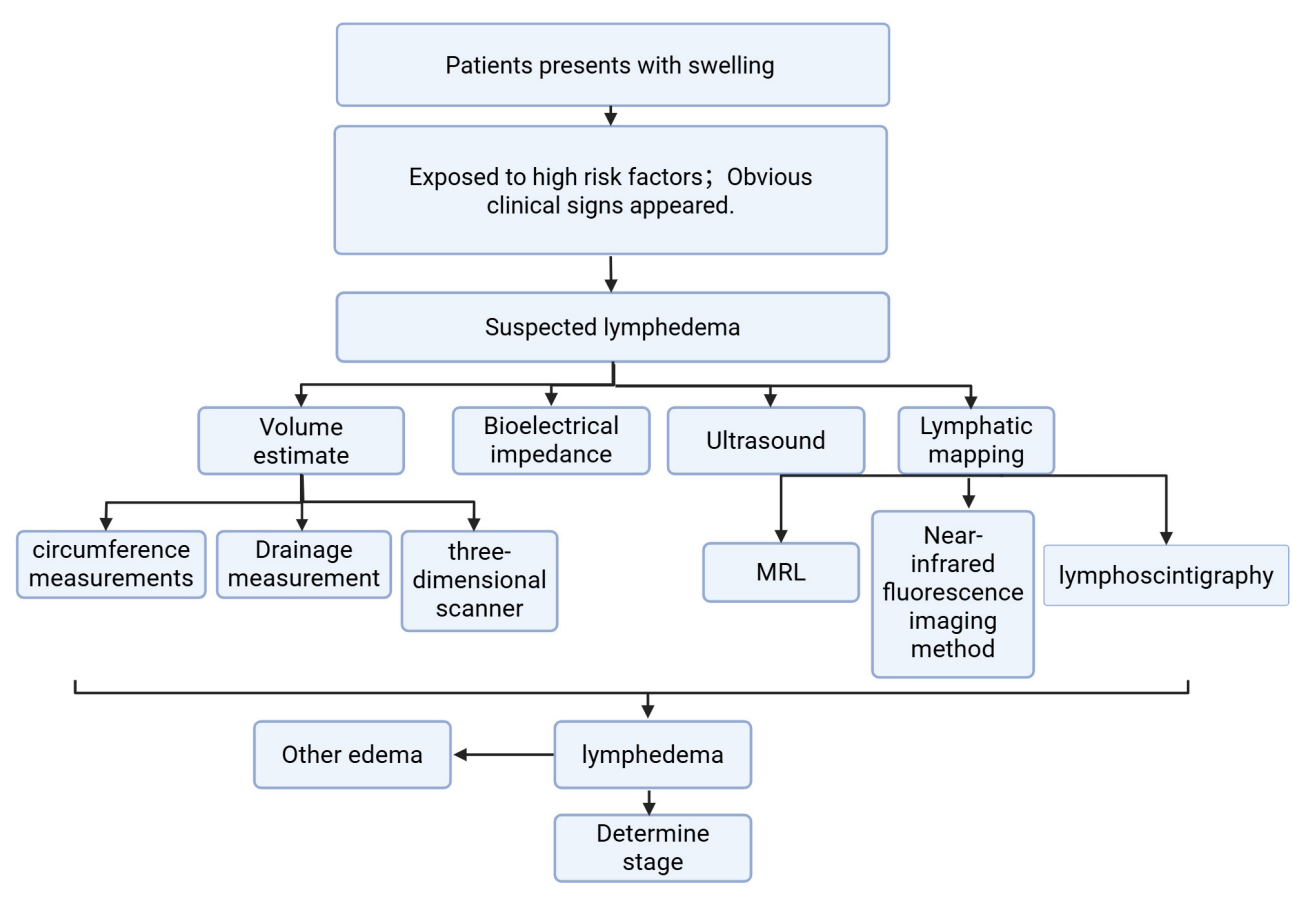
Diagnostic decision making flow chart for lymphedema.

In clinical practice, the selection of appropriate diagnostic methods for lymphedema depends on a variety of factors, including risk factors and physical examination findings. To illustrate the diagnostic decision-making process, Figure 1 provides a flowchart that integrates risk factors and objective limb measurements. This diagram outlines how clinicians can select the most appropriate diagnostic tests and interventions based on the patient's specific condition and presentation.

## 5 Clinical research and standard of care

Treatment of lymphedema aims to relieve symptoms, reduce swelling, and improve quality of life. Common treatment methods, including manual lymphatic drainage, total decongestive physiotherapy, compression sleeve therapy, exercise, and weight reduction, are all non-surgical approaches that require prolonged persistence. However, patient adherence to these treatments tends to decline over time. Surgical interventions such as lymphovenous anastomosis/bypass or vascularized lymph node transfer (VLNT), are applicable in early postoperative interventions and are ineffective for fibrosis caused by late lymphedema or fat deposition (99). Currently, no approved pharmacological standard therapy exists for lymphedema. However, early clinical data, such as the 2023 pilot study on topical tacrolimus, show promising results in reducing volume and symptoms (100). Additionally, translational research focusing on immune modulation (e.g., targeting the TGF-β axis and CD4+T cells) and fibrosis is emerging as a potential therapeutic approach (101). The mechanism of lymphedema is an important area of research to understand how lymphatic damage and interstitial fluid accumulation impair lymphocyte function and lead to a range of complications, which may help to identify appropriate targeted treatments for prevention and diagnosis. The establishment of collateral circulation pathways and the resolution of lymphatic circulation abnormalities are the core of the lymphedema treatment. Studies indicate that lymphatic side branches are present in both affected and absent limbs and are more frequent in primary lymphedema compared to secondary lymphedema (71). This is important for early diagnosis and treatment planning of lymphedema.

### 5.1 Non-surgical method: multiple methods are often used in combination

#### 5.1.1 Rehabilitation

The primary aim of non-surgical methods is to relieve the symptoms associated with swelling, rather than to cure the underlying disease. Complete decongestive therapy (CDT) is currently considered the standard treatment for lymphedema, including skin care, manual lymph drainage, compression and exercise. The duration of lymphedema is a predictor of treatment effectiveness (102). CDT can effectively reduce lymphedema, but the contribution of each component of complete decongestive therapy has not been determined. As one of the methods, Manual lymphatic drainage (MLD) may be beneficial in mild lymphedema and prevention, but its role in reducing tissue edema and extracellular fluid or skin thickness and improving fibrosis needs to be further explored. Long-term self-management is a challenge for patients with lymphedema, and CDT is not suitable for patients who have difficulty controlling blood pressure, paralysis, diabetes, bronchial asthma, acute infection, heart failure, and deep vein thrombosis (103). The majority of patients with primary lymphedema are controlled by compression therapy (compression bandages and compression garments) (104), exercise, and maintaining normal weight (105). For patients with secondary lymphedema, other modalities are usually more effective in combination with CDT. For example, the integration of electrotherapy modalities, specifically faradic current or ultrasound, in conjunction with CDT, has the potential to provide more substantial decreases in lymphedema volume, discomfort, and functional handicap (106).

In addition, there are many ways to further control the progression of lymphedema and reduce the impact of complications. In particular, electric stimulation (ES) has an influence on lymphedema's critical stages from onset to ulcer formation, inhibiting lymphedema progression and managing complications (107). The effectiveness of photobiomodulation (PBM) in treating head and neck lymphedema (108) and its anti-inflammatory and antifibrotic effects (109). Intermittent pneumatic compression (IPC) therapy and low-level laser therapy (LLLT) have been identified as efficacious interventions for the management of postmastectomy upper limb lymphedema (PML). When used together, these therapies have been shown to have slightly better long-term effects on pain compared to using pneumatic compression therapy alone (110).

#### 5.1.2 Pharmacotherapy

Inflammation preceded lipogenesis in the mouse tail lymphedema model, and inflammatory markers MCP-1 and nitric oxide may be potential targets for lymphedema management (111). Lymphedema and lymphatic stasis also led to CD4+ cell inflammation and mature T helper cell infiltration. CD4+ cell depletion significantly reduced lymphedema, inflammation, fibrosis, and fat deposition increased lymphangiogenesis, and reduced the pathological changes with lymphedema (112). Similarly, transcription of the type III collagen gene is also upregulated in fibrotic skin nodules of lower-extremity lymphedema (113). Therefore, inflammation-related pathological changes will provide a reliable treatment thought for lymphedema. Numerous studies have employed the migration and accumulation of CD4+ T lymphocytes in edematous regions as an innovative therapeutic target for addressing lymphedema (25, 112). Moreover, pharmacological therapies (Tacrolimus, Anti-IL-4/IL-13 antibodies, Leukotriene B4 antagonists) and cellular therapies have been demonstrated in animal models to treat lymphedema by promoting lymphangiogenesis, improving lymphatic function and inhibiting fibrosis and inflammatory responses (114, 115).

#### 5.1.3 Other treatments

Many cytokines also have potential in the treatment of lymphedema. T-cell-derived cytokines such as IL-4, IL-13, interferon-gamma (IFN-γ), and TGF-β1 are important negative regulators of lymphangiogenesis, which reduce collateral lymphatic vessel formation by inhibiting lymphatic endothelial cell (LEC) proliferation and lymphatic vessel formation, migration and function (26, 116). Inhibiting anti-lymphangiogenic cytokines to promote collateral lymphatic vessel formation is an important area of research. Compared to approaches that use pro-lymphangiogenic cytokines like VEGF-C or other methods, this approach reduces inflammatory responses without increasing cancer metastasis or tumor growth (117), which is a breakthrough in the direction of disease research. Transforming growth factor-β1 (TGF-β1) is an important regulator of extracellular matrix (ECM) deposition in secondary lymphedema, which is significantly increased in the skin of lymphedema patients. Inhibition of TGF-β1 in the mouse model of lymphedema can reduce extracellular matrix deposition, increase the formation of collateral lymphatic vessels, and inhibit the infiltration of T cells, which may play a role in the treatment of lymphedema (26). In recent years, cell therapy has also had a significant effect on lymphedema. Mesenchymal stem cells (MSCs) are believed to be progenitors of lymphatic endothelial cells with anti-inflammatory, anti-fibrotic, antioxidant stress, and immunomodulatory effects, which may be useful in promoting lymphangiogenesis to improve lymphedema (114). In addition, Toyserkani et al. (118) treated lymphedema for the first time using freshly isolated adipose-derived stromal cells and fat grafting, and patients had significant improvement in their daily symptoms after 4 months, reduced need for compression therapy, reduced volume of the affected arm, and no adverse events.

### 5.2 Surgical method: significantly improve symptoms

Surgical intervention utilizing reductive techniques is considered the preferred course of action for patients with lymphedema who have not experienced successful outcomes with conservative treatment methods. Suction-assisted liposuction effectively removes excess subcutaneous fibro-adipose tissue and may improve underlying lymphatic function (4) for the treatment of cancer-associated lower extremity lymphedema (119). However, it is important to emphasize that patients undergoing this procedure must wear compression garments for life to maintain the results and prevent recurrence. Additionally, factors such as gender, staging, and a previous history of recurrent dengue may impact the progression and outcomes of liposuction procedures (120).

Further innovations in microsurgical techniques, based on the use of indocyanine green to map lymphatic vessels during surgery, have improved the efficacy of lymphedema. Physiosurgical procedures are widely used, including LVA to divert lymphatic drainage into the veins, and VLNT to transfer healthy lymph nodes from unaffected areas of the body to lymphedematous limbs (74). LVA is a safe and effective method of reducing the severity of lymphedema, which correlates positively with the degree of lymphosclerosis and imaging stage, with lower extremity lymphedema being more severe and higher body mass index (BMI) and older age also leading to more severe lymphosclerosis (121). Hence, while LVA is most effective in managing early-stage upper extremity lymphedema, it can also benefit selected later-stage cases (122, 123). VLNT is believed to enhance the spontaneous regeneration of lymphatic vessel regeneration by stimulating the growth of new vessels from pre-existing capillary lymph vessels and lymphatic endothelial progenitor cells, which aids in the restoration of the regional lymphatic network, hence facilitating physiological recovery (124). VLNT is effective even in advanced cases and has been shown to reduce cellulitis incidence, as well as improve the impaired immunity associated with lymphedema (125, 126). Additionally, VLNT effectively reduces limb volume in both upper and lower limb lymphedema after cancer treatment (127). Nevertheless, the two approaches have little efficacy in addressing fat accumulation and lymphatic solidification and a firm and inflexible swelling during the advanced phase of the disease, therefore prompting the consideration of suction-assisted protein lipectomy (SAPL) as a potential alternative (128). However, it is important to note that patients who undergo SAPL are required to wear compression garments for life to maintain the results and prevent recurrence (129).

When faced with some complex situations, multiple methods are often used in combination. LVA combined with physiotherapy can reduce swollen volume, prevent cellulitis, and improve patients' quality of life (130). The utilization of a combined approach involving LVA and physiotherapy in conjunction with Lymphaticovenous anastomosis with node transfer (LVAN) is a highly efficacious treatment for both initial and advanced stages of lymphedema (126). Therefore, combination therapy should be used for advanced lymphedema (123).

### 5.3 Lymphedema prevention: a necessary way for this uncurable disease

Since lymphedema is hard to cure, and there will be many complications, it has a significant impact on the quality of life and morbidity of patients. Along with the substantial effort required to control it once it appears, the concept of lymphedema prevention is naturally attractive. Prognosis and preventive measures depend on the etiology and severity of the disease. For primary lymphedema, preventive measures mainly include regular self-management (lymphatic drainage massage, maintaining a healthy lifestyle, and avoiding injury or infection). For secondary lymphedema, it is mainly before and after surgery or radiotherapy to avoid the occurrence of lymphatic tissue damage or secondary infection. Radiotherapy can have an adverse effect on the outcomes of anastomosis by causing lymphatic fibrosis and impairing the regeneration of lymphatic vessels (131). At the same time, the update of technology also provides new possibilities for the prevention of lymphedema.

The timely identification of lymphedema, particularly among populations at high risk, is essential for effective preventative strategies. Comprehensively identifying and illustrating the potential risk factors predicting the occurrence of lymphedema are essential for the effective prevention and management of lymphedema (132). Near-infrared fluorescence lymphatic imaging (NIRF-LI) monitoring can characterize the early onset of peripheral lymphatic dysfunction as a predictor of breast cancer-associated lymphedema (133). Newer technologies such as bioimpedance spectroscopy (BIS) have shown the ability to detect subclinical lymphedema, allowing for early intervention and lower incidence of long-term lymphedema. Notably, the PREVENT trial demonstrated that BIS-triggered intervention significantly reduced the progression to chronic lymphedema compared to traditional tape measurement techniques (134).

Self-management is a viable strategy for the prevention of many issues (135). For instance, the use of self-management practices has a critical role in the prevention and management of lymphedema associated with breast cancer. Nevertheless, it is worth noting that breast cancer survivors exhibited inadequate lymphedema self-management practices. This deficiency can be attributed to their limited understanding of lymphedema, low levels of self-confidence, distorted perceptions of their condition, and insufficient social support. These factors should be taken into account when designing comprehensive intervention programmes (136). Multiple randomized clinical trials have provided evidence of the preventive efficacy of physiotherapy in the immediate postoperative period (137–139). While MLD has been shown to have a positive preventive impact on lymphedema (140, 141), its long-term efficacy in preventing the development of lymphedema may be limited (142). Thus, additional prophylactic strategies may be necessary.

Lymphedema Prevention Surgery (LPS) like axillary reverse mapping (ARM), immediate lymphatic reconstruction (ILR) Simplified Lymphatic Microsurgical Preventing Healing Approach (SLYMPHA) to preserve and restore lymphatic flow through lymphatic venous bypass (LVB), and has been shown to have the potential to reduce the risk of lymphedema in breast cancer patients requiring axillary lymph node dissection (143–145). Recent randomized controlled trial (RCT) data published in 2023 have demonstrated that ILR significantly reduces the incidence of BCRL following axillary lymph node dissection (ALND). This preliminary evidence highlights the promising role of ILR in preventing the development of lymphedema in these patients. However, the study's limitations include a relatively small sample size and short follow-up duration, which may affect the long-term applicability of the results (146). Lymphedema is observed in around 30% of breast cancer patients who have undergone axillary lymph node dissection. The implementation of ILR has been found to significantly decrease the risk of developing lymphedema. This is achieved through the construction of prophylactic lymphovenous anastomosis, which involves connecting disrupted lymphatic channels in the arm to nearby axillary venous tributaries after ALND (147), thereby providing a route for the restoration of lymphatic drainage (148). Based on the present findings, the utilization of ILR demonstrates considerable potential as a secure strategy for the prevention of lymphedema among patients at high risk (149, 150). Also, in the face of BCRL, axillary reverse mapping (ARM) was developed to map and preserve arm lymphatic drainage during ALND (151), which is a simple and effective technique that appears to have reduced lymphedema rates after axillary surgery (152, 153). SLYMPHA can be regarded as an adjunct procedure to ALND for all patients during breast surgery. It is a simplified version of LYMPHA. The study showed that the lymphedema rate of patients who underwent SLYMPHA was significantly lower in comparison to those without SLYMPHA (154).

The immune functions exhibit a notable decrease in individuals with secondary lymphedema, accompanied by an upregulation of many T-cell-associated networks in such circumstances. Furthermore, it has been demonstrated that lymphatic dysfunction plays a role in promoting secondary bacterial and fungal infections, as well as initiating inflammation in the skin and subcutaneous tissue, contributing to the advancement of lymphedema. Therefore, CD8+T-cell exhaustion patterns should be taken into consideration for the prevention of lymphedema (155). The study found that CD4+T cells contribute to lymphangiogenesis are activated in regional lymph nodes and migrate to the skin to initiate lymphedema which demonstrates that the CD4+T cell is a potential therapeutic target for the prevention of lymphedema (112, 116, 156). As shown in Table 6, the clinical evidence for the main treatment methods in lymphedema management is summarized.

**Table 6:** Clinical evidence table of main treatment methods.

**Intervention**	**Patient selection criteria/indications**	**Primary endpoints/outcomes**	**Follow-up duration**	**Limitations**
Complete decongestive therapy (CDT) (206–210)	Head and neck lymphedema; early to moderate lymphedema; compliant patients	Volume reduction, symptom relief, improved QoL; questionnaire for lower limb lymphoedema	5–18 months	Requires lifelong adherence; not suitable for patients with comorbidities (e.g., DVT, heart failure); supplemented with advanced treatments like liposuction or enhanced compression techniques in later stage; psychological support
Manual lymphatic drainage (MLD) (138, 211)	Mild lymphedema or prophylaxis	Subjective symptom improvement, reduced swelling; volumetric changes, adverse events, function, subjective sensations, QoL, cost of care	4–6 months	Limited evidence for long-term efficacy; operator-dependent
Lymphaticovenous anastomosis (LVA) (212–215)	Early-stage lymphedema; functional lymphatic vessels	Volume reduction, limb/subfascial volume, decreased cellulitis episodes	1–5 years	Less effective in late-stage fibrosis; requires microsurgical expertise
Vascularized lymph node transfer (VLNT) (216, 217)	Moderate to advanced lymphedema; failed conservative therapy	Volume reduction, improved lymphatic function, reduced infections; improved circumferential reduction rates and lymphedema-specific quality-of-life questionnaire scores	2–5 years	Donor-site morbidity; variable graft survival
Liposuction (SAPL) (119, 218, 219)	Non-pitting edema; fibrofatty deposition; advanced lymphedema	Volume reduction, improved contour	3–24 months	Requires lifelong compression; not addressing lymphatic function; sex, stage, and recurrent erysipelas history influence effect
Topical tacrolimus (100, 220, 221)	Early inflammatory lymphedema; women with stage I or II BCRL	Volume reduction, skin softening, improved arm volume, L-Dex, and HRQoL	3–12 months	Limited clinical data; not yet standard therapy
Exercise + weight control (222–225)	All stages, especially obese patients	Volume stabilization, improved mobility	3–12 months	Adherence challenging

## 6 Conclusion

Lymphedema reflects the convergence of genetic susceptibility, lymphatic injury, chronic inflammation, and tissue remodeling. Early detection and timely intervention are critical, as delayed treatment permits irreversible fibroadipose changes that diminish therapeutic efficacy. Current management combines careful risk assessment, advanced imaging, and multimodal therapy incorporating conservative, surgical, and emerging pharmacologic strategies. Future priorities include comprehensive genomic profiling of primary lymphedema, refinement of imaging biomarkers for preclinical disease, and development of targeted immunomodulatory and antifibrotic agents. Progress will depend on close integration of basic, translational, and clinical research to disrupt the pathogenic cycle, personalize therapy, and reduce the global burden of this disabling condition.
